# Morphometric and Microstructural Changes During Murine Retinal Development Characterized Using In Vivo Optical Coherence Tomography

**DOI:** 10.1167/iovs.62.13.20

**Published:** 2021-10-26

**Authors:** Simon Brais-Brunet, Émilie Heckel, Udayakumar Kanniyappan, Sylvain Chemtob, Caroline Boudoux, Jean-Sébastien Joyal, Mathieu Dehaes

**Affiliations:** 1 Institute of Biomedical Engineering, University of Montréal, Montréal, Canada; 2 Research Center, CHU Sainte-Justine, Montréal, Canada; 3 Department of Pharmacology, University of Montréal, Montréal, Canada; 4 Department of Pediatrics, University of Montréal, Montréal, Canada; 5 Department of Ophthalmology, University of Montréal, Montréal, Canada; 6 Department of Engineering Physics, Polytechnique Montréal, Montréal, Canada; 7 Department of Radiology, Radio-oncology and Nuclear Medicine, University of Montréal, Montréal, Canada

**Keywords:** retina, development, microstructure, wild-type mouse, optical coherence tomography (OCT)

## Abstract

**Purpose:**

The purpose of this study was to develop an in vivo optical coherence tomography (OCT) system capable of imaging the developing mouse retina and its associated morphometric and microstructural changes.

**Methods:**

Thirty-four wild-type mice (129S1/SvlmJ) were anesthetized and imaged between postnatal (P) day 7 and P21. OCT instrumentation was developed to optimize signal intensity and image quality. Semi-automatic segmentation tools were developed to quantify the retinal thickness of the nerve fiber layer (NFL), inner plexiform layer (IPL), inner nuclear layer (INL), and the outer retinal layers (ORL), in addition to the total retina. The retinal maturation was characterized by comparing layer thicknesses between consecutive time points.

**Results:**

From P7 to P10, the IPL increased significantly, consistent with retinal synaptogenesis. From P10 to P12, the IPL and ORL also increased, which is coherent with synaptic connectivity and photoreceptor maturation. In contrast, during these periods, the INL decreased significantly, consistent with cellular densification and selective apoptotic “pruning” of the tissue during nuclear migration. Thereafter from P12 to P21, the INL continued to thin (significantly from P17 to P21) whereas the other layers remained unchanged. No time-dependent changes were observed in the NFL. Overall, changes in the total retina were attributed to those in the IPL, INL, and ORL. Regions of the retina adjacent to the optic nerve head were thinner than distal regions during maturation.

**Conclusions:**

Changes in retinal layer thickness are consistent with retinal developmental mechanisms. Accordingly, this report opens new horizons in using our system in the mouse to characterize longitudinally developmental digressions in models of human diseases.

The study of the physiological development of the retina and its maturation is critical to characterize healthy growth but has been limited by the lack of appropriate in vivo longitudinal imaging instruments. Among markers of maturation,^1^^–^^9^ the analysis of retinal morphometry and retinal thickness over time, as well as more subtle changes of the microstructure of retinal layers, may help inform retinal development. In the mouse pup model, the in vivo quantification of retinal thickness profiles may be challenging due to the presence of hyaloid vessels in the vitreous and because the mouse lens and cornea are not fully developed. The developing retina undergoes neurovascular changes associated with maturational processes, including synaptogenesis, angiogenesis, and vascular remodeling, as well as cell differentiation, migration, and densification, which contribute to the complexity of in vivo retinal layer identification and classification. These challenges may explain the low number of in vivo imaging studies that explored retinal morphometric and microstructural changes during maturation.

Optical coherence tomography (OCT) is a noninvasive imaging modality used to characterize the morphology and the microstructure of the retina in both humans and animals.^10^^,^^11^ The primary source of OCT signal in biological tissue is the one that is detected by back-scattering light reflectance. In the retina, detected OCT intensity is precisely related to the cellular microstructure of the specific layers when light is emitted in the anterior-posterior direction. In particular, the hyper-reflective OCT signal measured in the nerve fiber layer (NFL) is due to the axons of the ganglion cells that are horizontally organized in the tissue.^4^^,^^12^ This horizontal microstructure leads to higher back-scattering reflected signals. Hyper-reflective signals are also measured in synaptic layers, such as the inner and outer plexiform layers (IPL and OPL, respectively).^13^ Theses synaptic regions are organized in layers. Amacrine, ganglion, and bipolar cells are connected in the IPL, whereas bipolar, horizontal, and photoreceptor cells are connected in the OPL. In contrast to synaptic layers, the inner and outer nuclear layers (INL and ONL, respectively) are composed of axons and perikarya that are oriented parallel to the anterior-posterior axis. This meticulous structural alignment creates hyporeflective signals, as optical scattering in these tissues is lower.^14^^,^^15^ The inner and outer segments (IS and OS, respectively) of the photoreceptors are vertically aligned and located underneath the ONL. There is no consensus about the origin of the hyper-reflective signal measured by OCT in this region. The signal was originally assigned to the IS/OS junction^16^ and re-assigned by clinicians in the ellipsoid zone due to mitochondria.^17^^,^^18^ Recent works provided additional evidence about the IS/OS origin.^19^^,^^20^ The brightest OCT signals detected in the retina originate from the retinal pigment epithelium (RPE) and the Bruch's membrane complex. These tissues are compact, dense, and highly organized layers of epithelial cells. The high density of melanosome in the RPE and the horizontal organization of the Bruch's membrane favor back-scattering and reflectance, respectively, which maximizes OCT intensity.^18^^,^^21^ The choroid also generates OCT signals due to the presence of highly dense vascular networks. In addition, the contribution of blood vessels located in the IPL and OPL is lesser than that of the choroid due to their lower vascular density. However, these vasculatures are visible when performing OCT angiography.^22^^,^^23^

Based on this retinal microstructural a priori knowledge,^18^ previous studies have reported retinal thickness measurements in the wild-type adult mouse (age greater than or equal to postnatal [P] day 28) using OCT,^21^^,^^24^^–^^31^ magnetic resonance imaging (MRI),^32^^–^^35^ and histology.^29^^,^^31^^,^^36^^–^^41^ Using these techniques, retinal thickness ranged between 170 and 265 µm. Some of these studies have also reported a strong correlation between OCT thickness measurements and histology,^29^^,^^31^^,^^42^^,^^43^ although absolute thickness measurements sometimes differed.^25^^,^^44^ Such difference in thicknesses was attributed to higher variability in histological observations due to sample preparation,^39^^,^^45^ or caused by the denaturation of the tissue postmortem.^30^^,^^31^^,^^46^ Although there was no direct comparison between OCT and MRI in the living animal, a recent study reported that human retina/choroid thickness was significantly higher when measured by MRI compared to OCT.^47^ Thicknesses of individual retinal layers in adult mice were also reported through segmentation techniques. In particular, from the anterior to the posterior retina, the NFL measured 10 to 20 µm, the IPL was about 50 to 60 µm, the thickness of the INL was between 20 and 30 µm, the OPL was about 15 µm, the ONL was between 45 and 65 µm, whereas the IS/OS measured 35 to 45 µm (in a 12-hour light/dark schedule).^29^^,^^42^^,^^48^ The IS/OS were also measured in light- and dark-adapted conditions, which had a significant impact on their thicknesses among other biological aspects.^49^ Retinal thickness measurements were also reported in wild-type mice before adulthood (age less than P28) using OCT,^24^^,^^30^^,^^50^^–^^52^ MRI,^33^^,^^35^^,^^53^ and histology.^36^^–^^38^^,^^41^ From P7 to P21, retinal thickness varied between 176 and 265 µm. However, none of these studies systematically characterized in vivo morphological and microstructural changes occurring in segmented retinal layers at multiple time points from P7 to P21.

During development, retinal layers undergo precisely choreographed temporal maturation that governs retinal composition and its morphology. In the murine retina, the thickness of the region surrounding the optic nerve head (ONH) increases until P21, whereas the RPE and the ganglion cell layer remain relatively constant.^50^^,^^54^ The development of photoreceptors and their synapses in the OPL and IPL has been extensively characterized^1^^,^^3^^,^^55^ and may contribute to increasing the retinal thickness around the ONH.^3^^,^^55^^,^^56^ The OPL and photoreceptors are sometimes regrouped and form the outer retinal layer (ORL).^27^ Murine vascular development also occurs in the first 3 weeks of life. The choroid nourishes the outer retina, whereas the superficial (P1–P10), deep (P8–P12), and intermediate (P12–P20) vascular plexi irrigate the inner retina.^4^^,^^57^ Densification, autophagy, and selective apoptosis of neurons and vessels contribute to pruning and remodeling of the developing retina.^6^^,^^57^^–^^59^ However, our understanding of retinal neurovascular development has been mostly limited to static ex vivo observations. To our knowledge, only a few studies reported retinal thickness measurements in living animals prior to P14,^30^^,^^33^^,^^51^^–^^53^ and these studies were limited to a single time point or whole retinal thickness measurements.

We developed a spectral-domain OCT instrument capable of imaging the morphometry and microstructure of the developing wild-type mouse retina between P7 and P21. We further developed segmentation tools to quantify changes in retinal layer thicknesses over time. These normative data can then be used in longitudinal studies that will assess morphological and microstructural alterations involved in a variety of retinal diseases.

## Methods

### Animal Model and Preparation

Animal procedures were performed in compliance with the ARVO Statement for the Use of Animals in Ophthalmic and Vision Research and the Animal Care Committee at CHU Sainte-Justine, Canada. Wild-type mice (129S1/SvImJ; Jackson Laboratory, stock 002448) were maintained and bred in standardized conditions at the animal facility and exposed to a routine light (day) and dark (night) cycle of 12 hours. Both littermate females (*N* = 14) and males (*N* = 20) were considered. Prior to imaging, mice were brought in a normal illumination room (500 lux) after at least 4 hours of light exposure, limiting variability in IS/OS thickness due to light exposure.^49^ Mice were anesthetized with a mixed solution (10 µL/g) of ketamine (Vetoquinol, 100 mg/kg) and xylazine (Bayer; 20 mg/kg). When necessary, eyelids were manually opened or surgically removed to access the eye. Pupils were dilated with drops of 2.5% phenylephrine (Alcon) and maintained wet during imaging using NaCl 0.9%. A head holder (921-E, KOPF) was used when necessary, and body temperature was maintained at 37°C. Post-imaging, animals were euthanized using euthanyl solution (Bimeda-MTC) before being awake.

### Imaging Design and Instrumentation

**Figure 1.**
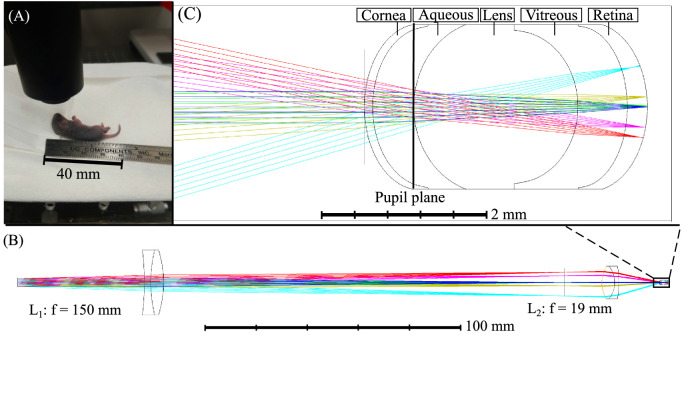
(**A**) Positioning of an animal of 7 days of age for non-contact retinal imaging under a custom-made quasi-telecentric lens system including two achromatic doublets (focal lengths of 150 and 19 mm, for L_1_ and L_2_, respectively). (**B**) Simulated beam rays propagating through the quasi-telecentric system. (**C**) Zoomed view of the beam rays in the animal eye with the beam focus plane located in the posterior retina.

Imaging was performed with a spectral-domain OCT system (Telesto 220; Thorlabs, Lübeck, Germany) emitting at 1300 nm with an axial resolution of 5.5 µm in air. A scanner head (OCTP-1300/M; Thorlabs) connected to the source and detection module was modified by integrating a custom quasi-telecentric lens system, including two achromatic doublets (focal lengths of 150 and 19 mm, for L_1_ and L_2_, respectively). OCT imaging was performed with no contact between the lens and eye (see the positioning of a P7 mouse under the lens in Fig. 1A). A magnification of 7.9 allowed to minimize the beam size at the pupil and maximized light intensity in the retina. The beam size at the mouse pupil was 400 µm. The system also allowed us to reduce the effective numerical aperture at the pupil and increased the Rayleigh beam range. This strategy allowed us to image the entire retina in depth. To further improve image quality, optimize the position of the lenses, and maximize the resolution of the beam in the mid-depth of the retina, numerical simulations were performed (Optic Studio Zemax, Kirkland, WA, USA) with parameters mimicking the anatomy of a P17 mouse eye.^41^^,^^60^ Figure 1B shows simulated beam rays propagating through the quasi-telecentric system, whereas Figure 1C is a zoomed view of the beam rays in the P17 animal eye with the beam focus plane located in the posterior retina. Additional simulations were performed to model the change in mouse eye size as a function of age. At the extremity of the field-of-view (i.e. for the worst focusing beam simulation parameters), the energy fraction within an area of a 7 µm radius was only slightly affected by the mouse eye size and ranged between 80.44 and 81.33% (see Supplementary Fig. S1). When considering the same beam angle for all time points, the position of the beam differed as a function of age, which impacted the field-of-view. The field-of-view ranged from 0.782 mm at P7 to 0.906 mm at P21. Image scales were then adjusted to compare all time points in the same reference space. The native axial scaling of 3.42 µm/pixel in air was corrected to 2.53 µm/pixel in tissue with a refractive index of 1.35.^61^

### Imaging Protocol

Thirty-four mice and 68 samples (eyes) were considered for imaging (see the Table). Specific time points included P7 (*N* = 6), P10 (*N* = 5), P12 (*N* = 5), P14 (*N* = 8), P17 (*N* = 5), and P21 (*N* = 5). Fourteen samples were excluded due to malformation, damage, or animal death. A 3D micrometric imaging platform was used to align the pupil with the light beam.^62^ This procedure ensured that the orientation of the retina was approximately identical for all animals; no biological feature, such as the macula, is available to create a reference system (quadrant).^63^ The field-of-view was centered around the ONH as a circular area of 0.5 mm radius. Data volumes of 512 BM-scans (B-scan over time) were acquired, each including 5 consecutive B-scans of 512 A-lines. An additional volume was acquired outside the field-of-view to quantify background noise.^64^ The imaging protocol included one OCT intensity volume and one OCT angiography (speckle variance) volume.^65^

**Table.** Number of Animals and Samples (Retinas) Per Time Point

Age Time Point, Postnatal (P) Days	Number of Animals	Number of Samples, Retinas
**P7**	6	8
**P10**	5	10
**P12**	5	7
**P14**	8	14
**P17**	5	7
**P21**	5	8
**Total**	**34**	**54**

### Image Processing and Analysis

The chromatic dispersion mismatch introduced by the eye was compensated numerically by adding a phase correction to the OCT spectral fringe pattern using second and third order dispersion coefficients that were calculated from a previous model.^66^ Corrections for uneven signal-to-noise ratio (SNR) roll-off and background subtraction were also applied.^67^ Two-dimension intensity-based image registration was performed to minimize eye movements, such as every B-scan was compared to the previous one, except the first one, which served as the initial reference.^68^

Structural images were obtained by intensity averaging, which reduced temporal signal fluctuations and increased SNR.^69^ Angiography images were obtained by calculating the variance of the signal intensity over time, which allowed for the detection of blood vessels and capillaries.^65^ To further reduce eye motion effects, the time frame inducing the highest variance was removed from the volume with a moving average technique.^68^ The resulting image corresponded to the lowest variance averaged over time.

### Segmentation Technique and Retinal Layer Assignment

**Figure 2.**
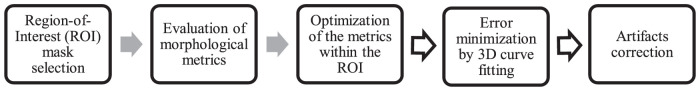
The workflow of the segmentation algorithm. Details of each step are provided in Supplementary Table S1.

To facilitate the detection of boundaries between retinal layers, convolution with a rectangular nearest-neighbor averaging kernel was applied to raw data. This procedure reduced static speckle noise to identify better each layer. Retinal layers were segmented with a custom-made, semi-automatic algorithm based on iterative spatial segmentation criteria (Fig. 2). Layer boundaries reported in previous studies were used as initial landmarks to perform segmentation.^30^^,^^70^^,^^71^ These criteria were based on the image intensity *I* and its axial derivative *D* (see details in Supplementary Table S1). In each iteration, an axial mask was applied to identify the layer boundaries. Before segmenting the first layer, the ONH was excluded using a semi-automatic algorithm searching for a disruption in an en face projection of the RPE and Bruch's membrane complex. The size of this disruption was used to define the ONH width size for each retina. The ONH identification in the posterior to anterior direction assumed a quasi-cylindrical shape with no curvature along this axis.

The RPE and Bruch's membrane complex are compact and dense tissues for which segmentation is challenging when considering OCT images.^17^^,^^18^ For the segmentation process, the two microstructures were considered as a single layer and termed RPE-Bruch complex. As a first step, the RPE-Bruch complex was identified as it provided the highest absolute OCT back-scattered intensity at 1300 nm (see retina depth in function of OCT signal intensity in Supplementary Fig. S2).^18^^,^^72^ Then, the NFL (that also provides hyper-reflective OCT signals at 860 nm)^71^ was identified with a first-derivative mask that detected the vitreous layer and a priori knowledge of the distance between the RPE-Bruch complex and the vitreous (approximately 200 µm).^26^ The OPL was identified by detecting a local maximum of *I* with a squared Hann filter.^73^ To identify the IPL-to-INL interface, a linear mask was applied on *D* from the vitreous posterior region to the OPL. Once the IPL posterior boundary was identified, a second Hann filter mask was applied on *D* to reveal the IPL anterior boundary. Then, the outer segment of the photoreceptor was detected by finding the maximum first derivative between the OPL and the first estimation of the RPE-Bruch complex. At P7, the separation of the nuclear region, the inner, and the photoreceptor (rod/cone) segments was impractical due to incomplete maturational stage.^3^^,^^55^ Thus, for all time points, all the segments of the photoreceptors (ONL, and the inner and outer segments) were combined in the ORL, as previously proposed.^27^

The robustness and reliability of the segmentation method was assessed by comparing semi-automatic retinal thickness measurements with manual measurements for all retinas using imageJ software.^74^ Four regions (one in each quadrant, 20 µm radius) located at 300 µm from the center of the ONH with no a priori knowledge about the presence of vessels were defined (see Supplementary Fig. S3). Bland-Altman and Pearson's correlation analyses showed a high correlation between the semi-automatic and manual techniques (*r* = 0.81) with a maximum difference of 20.0 µm (90% two-sided confidence intervals; Supplementary Fig. S4). A test-retest analysis showed that the maximum difference between the two techniques was similar to the first analysis (19.1 µm, 90% two-sided confidence intervals; Supplementary Fig. S5). In addition, semi-automatic retinal thickness measurements were compared to histological images at P7 (see Supplementary Fig. S6 and Fig. S7) using imageJ software. In addition, a third validation of the segmentation technique was performed with OCT data previously published and acquired in adult animals (6 weeks old).^25^ In particular, manual measurements of retinal thickness reported in this previous study were compared to semi-automatic measurements. None of these comparisons was significantly different.

### Artifacts Correction

Spatial deviations induced by shadow artifacts and punctual variations were corrected. In particular, layer boundaries were smoothed by a high-order polynomial fit. For every A-line, the difference in depth between the layer voxels and their boundary was evaluated, and the mean difference with the segmentation curve was identified. The voxels that were located more than two standard deviations from the curve were corrected at the depth position of the fitted curve.

### OCT Layer Thickness Measurement and Region-of-Interest Selection

Each layer thickness was measured by subtracting the depth of consecutive boundaries over the segmented area in a cylindrical region-of-interest (ROI) of an average of 51 µm radius and a standard deviation of 12 µm. For each retina, the ROI selection was performed using OCT angiography images. Each ROI was located in a region excluding major blood vessels from the NFL. The thickness for each layer was then averaged within samples for each time point. The total retinal thickness was defined as the region between the posterior part of the vitreous and the RPE-Bruch complex posterior boundary. A second selection of ROIs in all samples was performed to ensure the reproducibility of the results described below. Another set of concentric ROIs was defined to study maturational changes surrounding the ONH. These ROIs were defined by 55 µm bands located around the ONH (adjacent ROIs) and from the end of the imaging field-of-view (distal ROIs, approximately 0.4 mm from the ONH, depending on the ONH width and imaging field-of-view). For this analysis, three samples were removed due to regional overlays.

### Statistical Analysis

For each retina and its layers, a Kruskal-Wallis test was used to assess the statistical difference in thickness in any pair of two time points. Then, linear mixed models with repeated measurements were used to assess the significance level of these comparisons. A Tukey-Kramer correction was applied to account for multiple comparisons and a corrected *P* value (*p*_corrected_) < 0.05 was considered significant. For comparison between adjacent and distal ROIs, as well as with P7 histological samples, age was fixed in the linear mixed model due to its interaction with ROIs. Data points were considered outliers if their values were lying outside the interval (*q_1_* – 1.5 × [*q_3_* – *q_1_*], *q_3_* + 1.5 × [*q_3_* – *q_1_*]), where *q_1_* and *q_3_* are the 25th and 75th percentiles of the datasets, respectively. Outliers were used in statistical comparisons.

## Results

**Figure 3.**
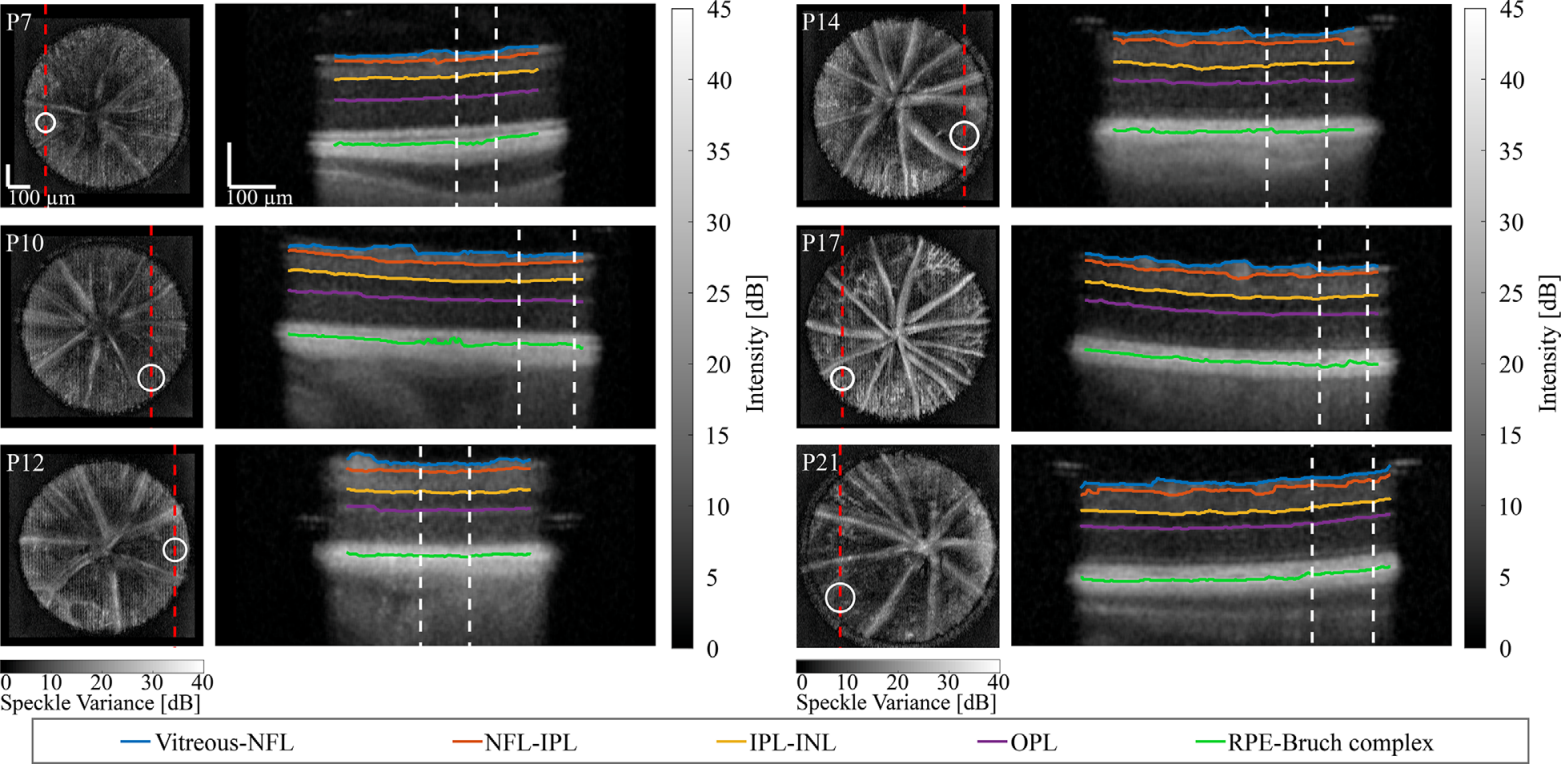
Regions-of-interest (ROIs, white circles) superimposed on optical coherence tomography (OCT) en face angiography views (left column) and associated segmented layer boundaries superimposed on depth views (right column) are shown at each time point (from postnatal [P] day 7 to P21). In particular, the vitreous and nerve fiber layer (NFL, blue), NFL and inner plexiform layer (IPL, orange), IPL and inner nuclear layer (INL, yellow), and combined retinal pigment epithelium (RPE)-Bruch complex and choroid (green) boundaries were identified and superimposed on OCT depth views. In addition, the outer plexiform layer (OPL, magenta) served as the boundary between the INL and outer nuclear layer (ONL). On en face images, the dashed red lines represent the 2D cross-sections. On depth images, ROIs are identified as the region between the dashed white lines. Scale bars are 100 µm.

Selected ROIs (white circles) superimposed on en face OCT angiography views (left column) and associated segmented layer boundaries superimposed on depth views (right column) are shown at each time point in Figure 3. In particular, the boundaries between the vitreous and NFL (blue), NFL and IPL (orange), IPL and INL (yellow), and RPE-Bruch complex and choroid (green) were identified. In addition, the OPL (magenta curve) served as the boundary between the INL and ONL. On en face images, the dashed red lines represent the 2D cross-sections. On depth images, ROIs are identified as the region between the dashed white lines. These segmentations were used to quantify the total retinal thickness and the thickness of each layer during maturation.

**Figure 4.**
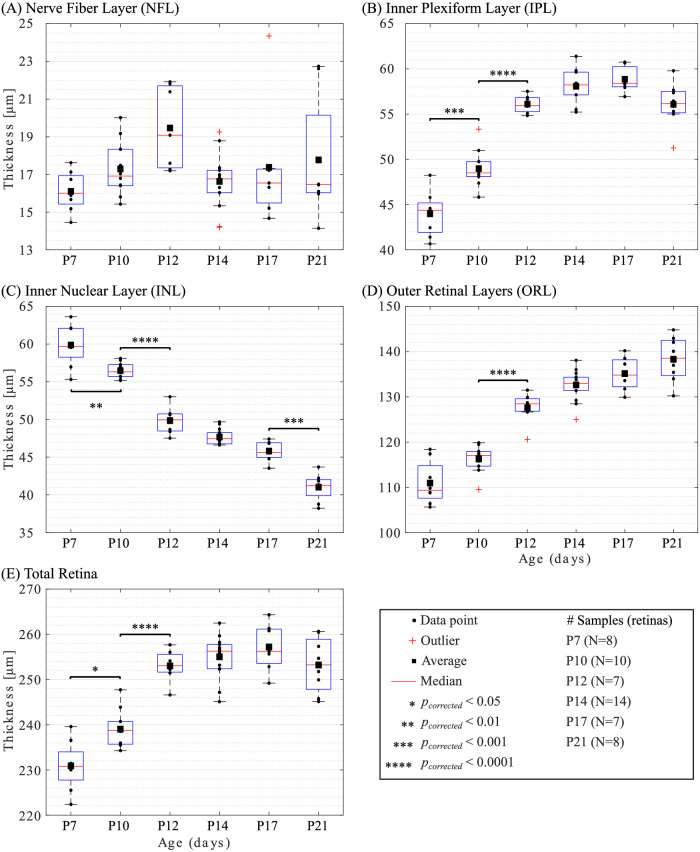
Optical coherence tomography (OCT) measurements of the retinal layers and total retinal thicknesses during maturation (from postnatal [P] day 7 to P21). For each time point, boxplots of all samples from the nerve fiber layer (NFL) (**A**), inner plexiform layer (IPL) (**B**), inner nuclear layer (INL) (**C**), outer retinal layer (ORL) (**D**), and total retina (**E**) are shown with the average, median, interquartile, and outliers.

OCT retinal layers and total retinal thicknesses as a function of age are displayed in Figure 4. Thickness profiles were measured in ROIs described in Figure 3. For each time point, boxplots of all samples from the NFL (A), IPL (B), INL (C), ORL (D), and total retina (E) thicknesses are shown with the average, median, interquartile, and outliers. From P7 to P10, the IPL increased significantly while the INL decreased significantly, which led to a significant increase in total retinal thickness. Between P10 and P12, the IPL and ORL increased significantly while the INL decreased significantly. These changes also led to a significant increase in total retinal thickness. From P12 to P14, the IPL and ORL trended slightly thicker while there was a trend toward a decrease in the INL and NFL. Between P14 and P17, the trend toward a decrease in the INL persisted while the other layers plateaued. From P17 to P21, the NFL and ORL plateaued while the other layers, together with the total retina, trended toward slightly thinner with a significant decrease in the INL. Overall, thickness changes in the total retina were attributed to changes in the IPL and ORL between P7 and P17 while in the INL between P17 and P21. The variability (standard deviation) for all layers at all time points was low (<7 µm), with the highest values observed for the total retinal thickness. Additional statistical comparisons between all time points and details on retinal layer thicknesses are provided in Supplementary Table S2 and S3, respectively.

**Figure 5.**
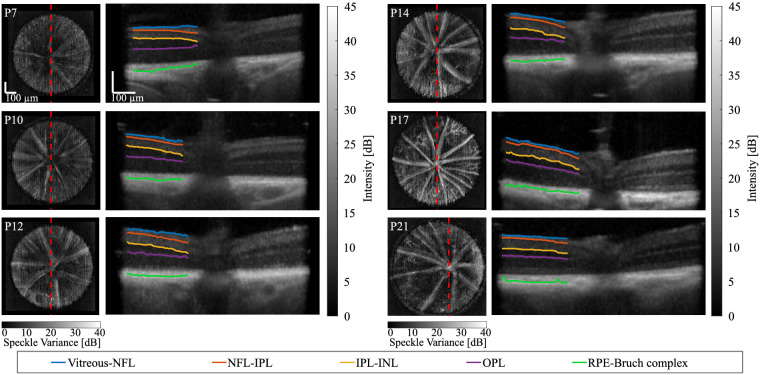
En face angiography views (left column) and associated segmented layer boundaries superimposed on depth views (right column) from the periphery of the retina to the optic nerve head (ONH), for each time point (from postnatal [P] day 7 to P21). In particular, the vitreous and nerve fiber layer (NFL, blue), NFL and inner plexiform layer (IPL, orange), IPL and inner nuclear layer (INL, yellow), and combined retinal pigment epithelium (RPE)-Bruch complex and choroid (green) boundaries were identified and superimposed on OCT depth views. In addition, the outer plexiform layer (OPL, magenta) served as the boundary between the INL and outer nuclear layer (ONL). On en face images, the dashed red lines represent the 2D cross-sections. On depth images, segmentations were only displayed on the left side of the ONH to compare with the nonsegmented morphology on the right side. Scale bars are 100 µm.

**Figure 6.**
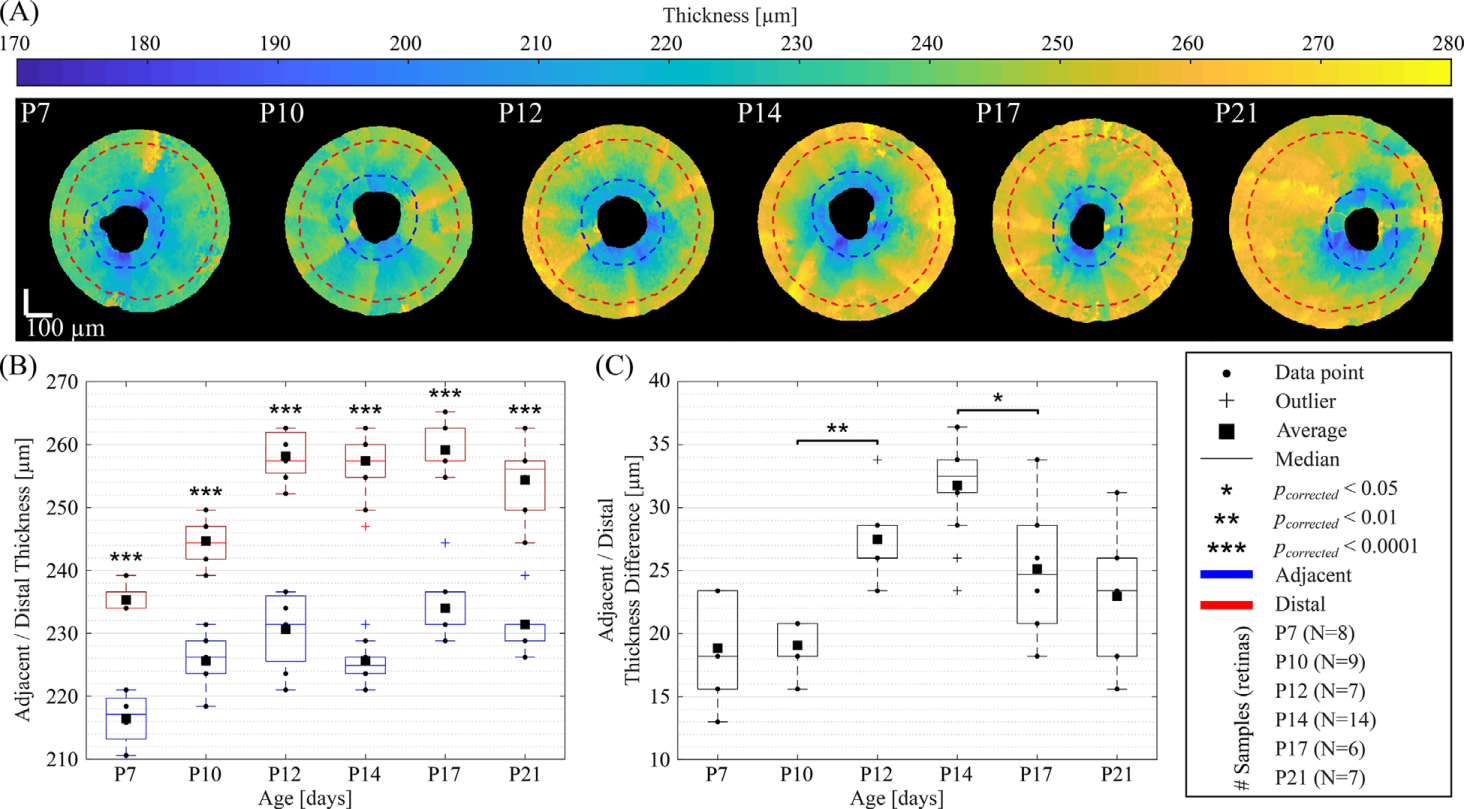
(**A**) En face views of the total retinal thickness for each time point (from postnatal [P] day 7 to P21), superimposed with ROIs adjacent (dashed blue lines) and distal (dashed red lines) to the optic nerve head. (**B**) Thicknesses of adjacent and distal regions as well as (**C**) their differences, all reported in boxplots representations including the average, median, interquartile, and outliers. Scale bars are 100 µm.

In Figure 5, we show en face OCT angiography views (left column) and associated segmented layer boundaries superimposed on depth views (right column) from the periphery of the retina to the ONH, for each time point. Segmentations were only displayed on the ONH's left side to visually compare with the nonsegmented morphology on the right side. On en face images, the dashed red lines represent the 2D cross-sections. These segmentations were used to characterize the microstructural changes of the total retina in both distal and adjacent regions to the ONH (see Fig. 6 for quantitation of these regions).

Figure 6A shows en face OCT views of the total retinal thickness for each time point, superimposed with ROIs adjacent (dashed blue lines) and distal (dashed red lines) to the ONH. The thickness of these adjacent and distal regions as well as their differences are reported in boxplots representations in Figure 6B and Figure 6C, respectively. Boxplot representations include the average, median, interquartile, and outliers (see legend). For all time points, the retina is significantly thicker in the distal regions compared to regions surrounding the ONH. In both distal and adjacent regions of the ONH, the retina thickened from P7 to P10 (*p_corrected_* < 0.01). In ROIs distal to the ONH, the retina continued to increase between P10 and P12 (*p_corrected_* < 0.001), reached a plateau between P12 and P17, and trended slightly thinner between P17 and P21. In ROIs adjacent to the ONH, the retina exhibited a similar evolution than in distal regions, except that the retina trended slightly thinner between P12 and P14, and then increased significantly between P14 and P17 (*p_corrected_* < 0.01). In addition, the difference between distal and adjacent regions plateaued from P7 to P10. The difference increased significantly between P10 and P12, and reached a peak at P14, before showing a significant decrease from P14 to P17.

## Discussion

This study highlights the in vivo capability of OCT to image the mouse retina as early as P7. Instrumental and algorithmic developments allowed us to characterize in vivo morphological and microstructural changes that occur during retinal development. By providing normative measurements in wild-type mice, this study provides comparative data for future work in disease conditions observed in the developing mouse.

During development, synaptic branching and connections increase in the IPL, consistent with synaptogenesis.^1^^,^^3^ This maturational process is reflected by the increase in the IPL thickness occurring before P12. This observation suggests increased connectivity before the natural opening of the eyelids occurring around P14.^55^ In contrast, the INL thickness profile decreases from P7 to P12 and further between P17 and P21. This observation is consistent with a previous study that reported cellular densification of the INL, which occurred concurrently with its thinning.^1^ In addition, thinning of the INL could also be the synchronous consequence of selective pruning observed during the nuclear migration of interneurons,^6^ which is not compensated by subsequent cell proliferation as reflected by a persisting decrease in thickness thereafter.

The outer retina (OPL, ORL, and RPE-Bruch complex) also undergoes morphological changes. The ORL thickness increases between P10 and P14. This increase is consistent with histology, which shows that photoreceptors elongate during maturation in preparation for light exposure.^3^ The thickening of the ORL continued to increase by P21 slightly. Consequently, the thickening of the total retina seems guided by the growth of the ORL and IPL between P7 and P12. In contrast to early development (P7–P12), the period between P17 and P21 reveals a slight decrease in the total retinal thickness, which may be attributed to a significant decrease in INL thickness likely driven by cellular densification, autophagy, and apoptosis. The trend in the total retinal thickness to decrease at P21 is also consistent with previous data, where the total retinal thickness becomes thinner from 260 µm at around P20^50^^,^^51^ to 200 to 220 µm months later.^28^^–^^30^^,^^51^^,^^75^ The validation of our technique with histology at P7 and manual measurements between P7 and P21, and at 6 weeks of age^25^ demonstrate the capability of our technique to capture morphometrical and microstructural changes in vivo during physiological retinal development. However, its capability to segment retinal layers in murine models of retinal degeneration remains to be validated.

At P7, the OPL begins to take shape but its detection on OCT was less challenging at P10. The increase in OCT signal is consistent with the previously observed OPL synaptic growth around P10.^3^ This increased OCT signal was also observed in the IPL. The ability to characterize with OCT early changes (P7–P10) in layers critical to synaptogenesis might help inform the pathogenesis of developmental diseases.

In regions excluding major blood vessels, the NFL thickness showed no significant change during retinal development. This observation is consistent with a previous study describing retinal development with light microscopy.^54^ Because the NFL is mostly composed of non-myelinated axons lying horizontally on the retina's surface, its nerve fibers do not thicken over time nor do they proliferate, as is expected from differentiated nerve cells. Although there is no significant change in the NFL thickness from P7 to P21, it is expected to observe local structural changes resulting from angiogenesis and occurring from P0 to P30.^4^^,^^57^ The first phase of postnatal vessels growth is characterized by large vessels that develop radially from the ONH to the periphery within the NFL (P0–P4). From P4, vessels develop in a laminar network toward the deep (P10) and then the intermediate (P30) plexus. From P7, a remodeling process occurs in the NFL with the formation of a capillary network from radial vessels that reduce in length and number of branches, and an increase in the distance between vessels.^57^ These results were observed with ex vivo fluorescence microscopy. In our study, the quantification of vascular density in the intermediate and deeper plexus was limited by weak SNR, in particular at P7. In the NFL, it was also challenging to infer if the vessels were part of the first phase of growth or resulting from ongoing remodeling starting at P7. Additional technical and methodological modifications of the current imaging system might allow us to study in vivo angiogenesis.

Adjacent regions to the ONH were thinner compared to distal locations. Between P10 and P12, this difference increased while decreasing between P14 and P17. This thickness difference may be related to the ONH's anatomy, which is supported and held in place by the *astrocytic lamina* and the peripapillary sclera that creates a biomechanical tension.^76^^,^^77^ The vascular regression of hyaloid vessels during early development may also contribute to the difference in regions adjacent and distal to the ONH.^78^ In addition, specific maturational processes occurring at the cellular and vascular levels of the retinal layers may also lead to thickness changes in regions distal and adjacent to the ONH. Through the entire retina, angiogenesis and vascular remodeling produce changes in the vascular network density and distribution of vessels.^4^^,^^57^ In the OPL and INL, synaptogenesis and neural differentiation occur during retinal development, in particular between P0 and P14.^55^ From P12, cellular densification was observed in the INL while the thickness of the IPL increased.^1^ Cellular differentiation was also observed concurrently with phagocytosis in the plexiform layers and the posterior region of the retina.^79^^,^^80^ With apoptosis,^81^ these natural forms of cell death affect the composition of the retinal layers, which may lead to changes in their thickness during development.

Performing noninvasive in vivo OCT imaging in the small and young animal retina introduced experimental challenges, including the necessity of opening the eyelids manually in some animals aged between P7 and P14. This intervention may affect the typical development of the eye and limits the longitudinal capability of the method, but this inference needs to be confirmed. The OCT signal was reduced between P7 and P14 compared to later time points. The OCT signal decreased as a function of pupil size, which limited SNR and image quality. However, the optical lens was designed to reduce the size of the light beam entering the vitreous, which minimized the impact of the small aperture. The reduction of the beam size further led to a larger beam waist at the retina, which limited the optimal lateral resolution, but ensured that the entire thickness of the retina was included in the Rayleigh zone. In addition to the small pupil size, the immaturity of the murine lens, the ocular shape, and hyaloid vessels also caused refractive errors, which include myopia or hyperopia, impacting lateral image resolution. These anatomic limitations were compounded by the spherical aberration associated with the small radius of the eye curvature, which also limited signal intensity. Although the reduction of the beam size minimized the impact of low signal intensity, it required enlargement of the beam spot size at the focal point, which further deteriorated spatial resolution. Low SNR also affected angiography between P7 and P14, and only large vessels were detected in the NFL. At P7, the optical aperture size is about 1.5 mm which led to lower quality angiography.^41^ The image quality increased through maturation as the aperture size increased with the size of the eye (estimated to 1.74 mm at P21).^41^ Finally, the OCT signal was hindered by the hyaloid vessels that are still present at P7; we imaged the retina using a longer light wavelength (1300 nm), which is less scattered by hyaloid vessels than a shorter wavelength.

Image quality may also be affected by the position of the focusing beam plane in the retina. The difference in image quality between ages was partially due to the positioning of the focusing beam plane, which was optimized for the P17 mouse and placed in the mid-depth retina for imaging. For younger and older mice, the focusing plane may be slightly repositioned in anterior or posterior layers of the retina to optimize image quality.

Technical challenges included the development of a quasi-telecentric scanning lens for P7 imaging. A custom reference arm was built with lenses that matched the sample arm. These lenses matched the dispersion induced by the lenses in the sample arm and followed a quasi-telecentric arrangement to allow a reflection of the collimated beam onto a retroreflector. Because of water absorption, emitting at 1300 nm also affected the light propagation in the eye and led to signal drops. However, the short pathlength and the low dispersion in water used to moisturize the eye further limited the impact on this optical mismatch.^82^ In addition, no water cell was added in the reference arm and the remaining optical mismatch was corrected numerically.^66^ Image artifacts and signal drop further affected segmentation results. In particular, similarities between morphological features in the NFL-IPL and IPL-INL boundaries limited the performance of the algorithm to reliably differentiate the layers. To remove this ambiguity, the algorithm was designed to detect the NFL-to-IPL interface by applying a linear mask from the posterior region of the vitreous, which made the automatic delineation more reliable.

Another useful technique to evaluate retinal thickness in living animals is MRI.^83^ Previous studies have used high-field MRI to quantify retinal thickness in wild-type mice.^32^^,^^33^^,^^35^^,^^53^ In particular, the retinal thickness of C57Bl/6J mice measured 241 ± 19 µm (*N* = 6) at P23 to P26.^35^ This study also reported a mean IPL thickness of 60 ± 9 µm and 40 ± 5 µm for the INL at P23 to P26. With a slightly different timing of measurements, these results are consistent with our data at P21. In adult mice (2–3 months of age), the majority of previous OCT and MRI studies agree on total retinal thickness measurements, although the latter tends to yield thicker measurements (around 20 µm).^28^^,^^29^^,^^33^^,^^53^^,^^75^^,^^84^^,^^85^ In addition, other MRI studies have reported total retinal thickness measurements in P7 wild-type mice (between 249 and 257 µm)^33^^,^^53^ that also slightly differ by approximately 22 µm from the ones reported here (231 µm). A similar overestimation was also previously reported in humans.^47^ This slight discrepancy may potentially be due to the limited MRI in-plane resolution (approximately 22–23 µm) and the application of partial volume averaging to estimate the retinal thickness. This technique led to a minimal choroidal contribution to the measurement.^53^^,^^86^^–^^88^ This limitation is less likely to occur with our technique as the axial resolution (in depth) is much lower (2.53 µm).^83^ Future research is needed to confirm the origin of this disagreement.

In contrast to the MRI study,^33^ the increase in retinal thickness prior to P14 showed in our data is consistent with previous ex vivo retinal thickness measurements performed within a similar temporal interval (P7–P21).^54^ Our data and previous studies^54^^,^^89^ also showed that the physiological pruning of the retina in wild-type mice is initiated between P14 and P21, which is not in agreement with studies showing that the retinal thickness decreases from P6 to P7.^33^^,^^37^ Although a previous OCT study showed an exponential decay of the retinal thickness from P14 to P206, local changes in retinal thickness within P14 to P21 (the period that overlaps our time points) were not significantly different and showed a plateau.^30^ Finally, slight differences between in vivo measurements of the retinal thickness may also be partially due to other factors, including a different animal strain^26^^,^^90^^,^^91^ and inter-strain variability,^38^ although a previous MRI study did not observe these differences.^92^

The comparison between in vivo OCT retinal layer thickness measurements and ex vivo histological assessments was not performed in this study (except at P7 for validation of the segmentation tool). Histological analysis of retinal cross-section for mice pup aged under P14 often yields more variable retinal thickness measurements. Due to the small size of the eyes, using appropriate comparative landmarks between retinas is critical and sometimes challenging. Various tissue fixation protocols and sectioning techniques may influence thickness including layer-specific shrinkage, as previously reported.^30^^,^^39^^,^^44^^–^^46^ Some of these challenges are attenuated in adult murine models where good agreement was reported between OCT and histology measurements of layer thicknesses.^31^^,^^42^^–^^44^

Future works include the design of longitudinal in vivo protocols to study normal and pathological retinal development. To perform these experiments, tunable optical components will be developed to adapt the OCT system through maturation. Accordingly, these technological developments will allow us to study maturational digressions in mouse models of human diseases.

## Conclusion

In this study, we developed a novel noninvasive OCT approach to image morphometrical and microstructural changes in vivo during physiological retinal development. We designed a specific segmentation tool that allowed us to differentiate retinal microstructures from 7 to 21 days of age and quantify retinal layer thickness profiles, as well as total retinal thickness in regions distal and adjacent to the ONH. Our observations are consistent with maturational mechanisms that were previously reported through ex vivo techniques. Our technique has the potential to be used to study retinal developmental diseases. The present study provides normative data for future comparisons.

## Supplementary Material

Supplement 1

Supplement 2

Supplement 3

Supplement 4

Supplement 5

Supplement 6

Supplement 7

Supplement 8

Supplement 9

Supplement 10
