# Supplementary material for: Morphometric and Microstructural Changes During Murine Retinal Development Characterized Using In Vivo Optical Coherence Tomography
Source: Invest Ophthalmol Vis Sci. 2021 Oct 26;62(13):20. doi: 10.1167/iovs.62.13.20 (PMC8556565; doi:10.1167/iovs.62.13.20)
Supplement: Supplement 1 [file iovs-62-13-20_s001.pdf]

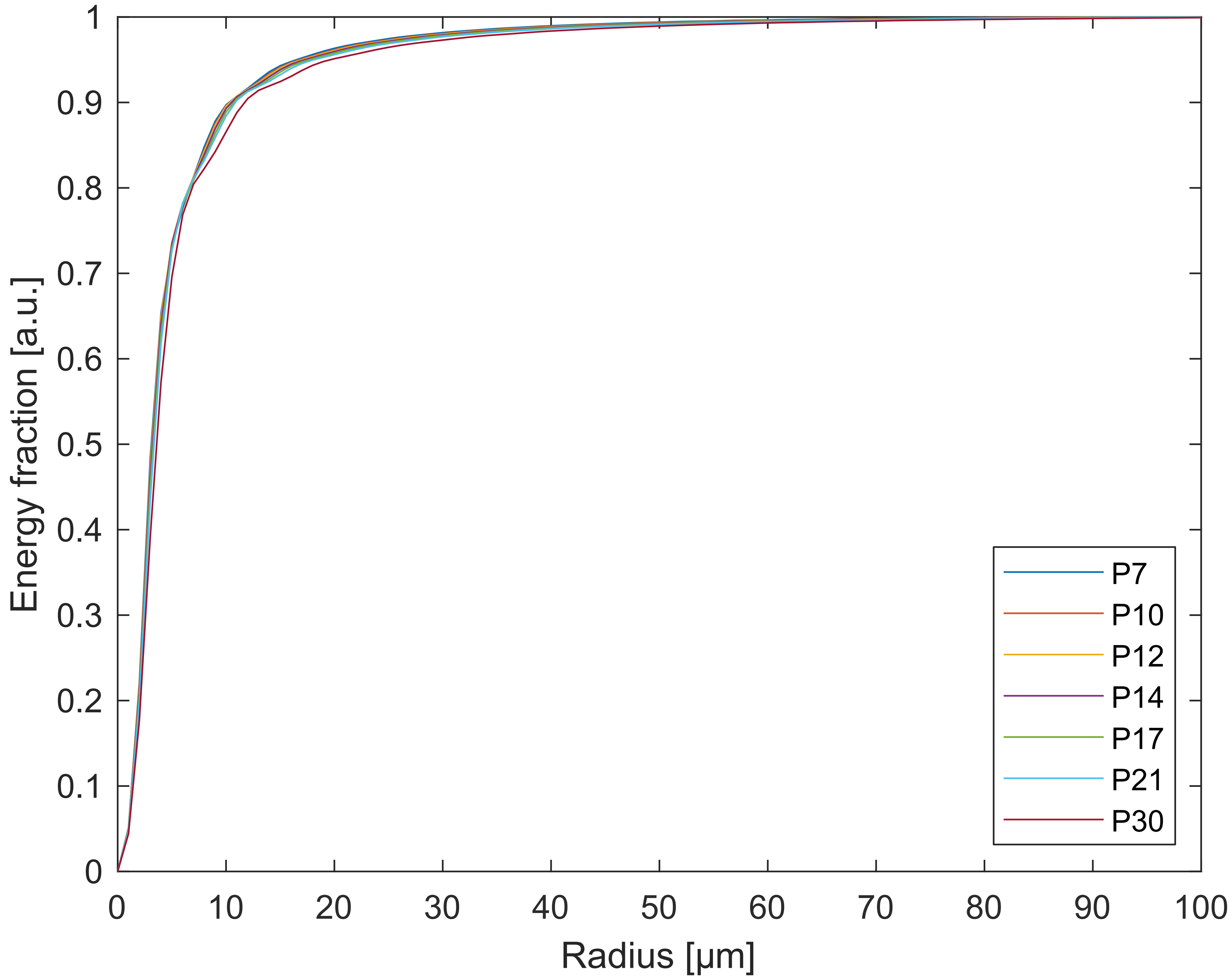

Supplementary Figure S1. Enclosed energy of the light beam at the retina and located at the extremity of the field of view for every time-point between postnatal (P) 7 and P30. Foreach time-point , at leat 80% ofthe energy is enclosed in a area of 7 µm radius.
