# Supplementary material for: Morphometric and Microstructural Changes During Murine Retinal Development Characterized Using In Vivo Optical Coherence Tomography
Source: Invest Ophthalmol Vis Sci. 2021 Oct 26;62(13):20. doi: 10.1167/iovs.62.13.20 (PMC8556565; doi:10.1167/iovs.62.13.20)
Supplement: Supplement 2 [file iovs-62-13-20_s002.pdf]

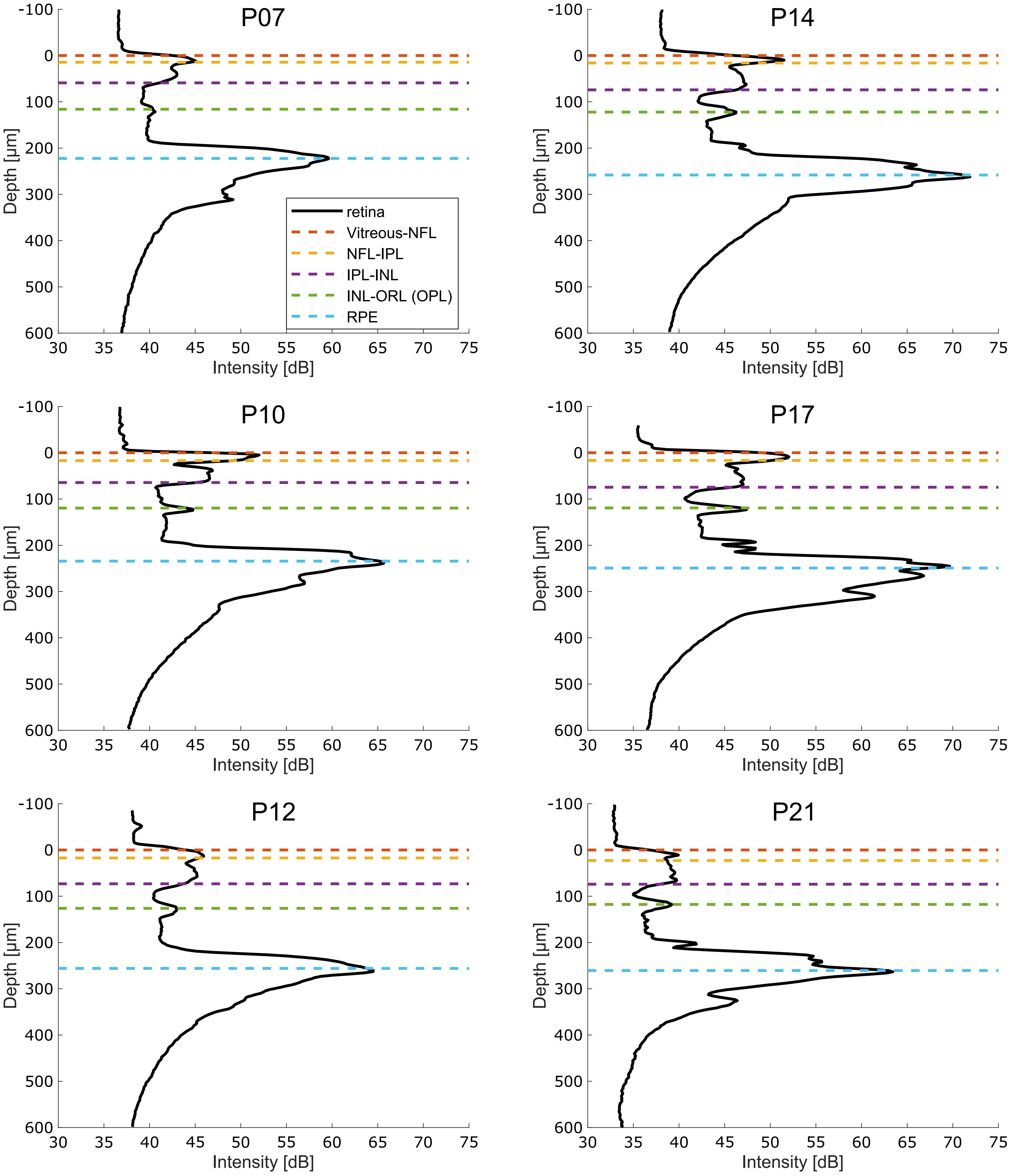

Supplementary Figure S2. Retina depth in function of OCT signal intensity used to perform retinal layers assignment. The two highest intensity peaks correspond to the retinal pigment epithelium (RPE)/Bruch's membrane complex and junction between the vitreous and the nerve fiber layer (NFL).
