# Supplementary material for: Morphometric and Microstructural Changes During Murine Retinal Development Characterized Using In Vivo Optical Coherence Tomography
Source: Invest Ophthalmol Vis Sci. 2021 Oct 26;62(13):20. doi: 10.1167/iovs.62.13.20 (PMC8556565; doi:10.1167/iovs.62.13.20)
Supplement: Supplement 3 [file iovs-62-13-20_s003.pdf]

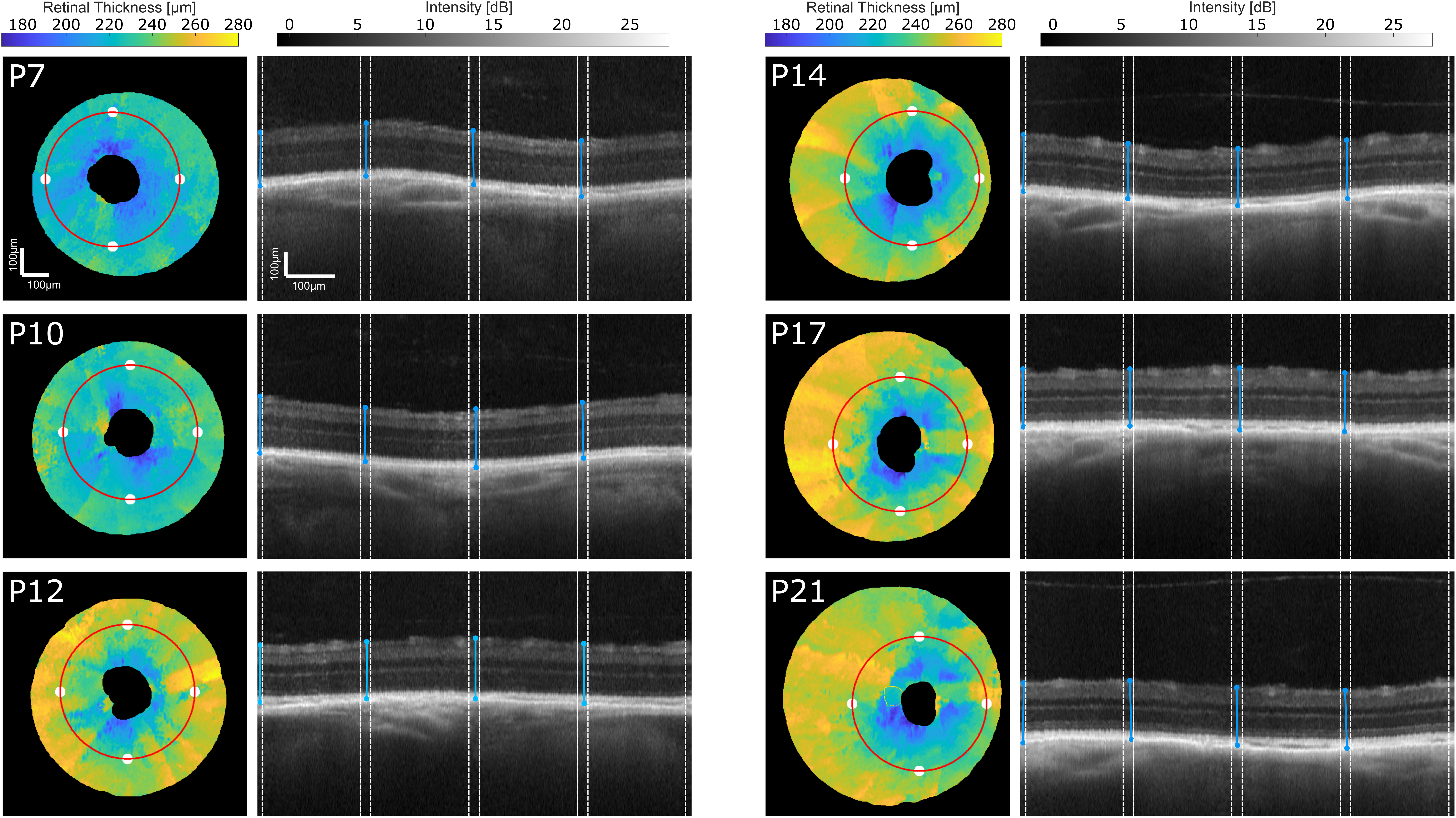

Supplemetary Figure S3. Systematic measurements of retinal thickness in four regions (white full circles, 20 µm radius, en face views) located at 300 µm (red ring) from the center of the ONH (left image for each time-point). Depth views showing the manual measurements (blue lines, right image for each time-point).
