# Supplementary material for: Morphometric and Microstructural Changes During Murine Retinal Development Characterized Using In Vivo Optical Coherence Tomography
Source: Invest Ophthalmol Vis Sci. 2021 Oct 26;62(13):20. doi: 10.1167/iovs.62.13.20 (PMC8556565; doi:10.1167/iovs.62.13.20)
Supplement: Supplement 4 [file iovs-62-13-20_s004.pdf]

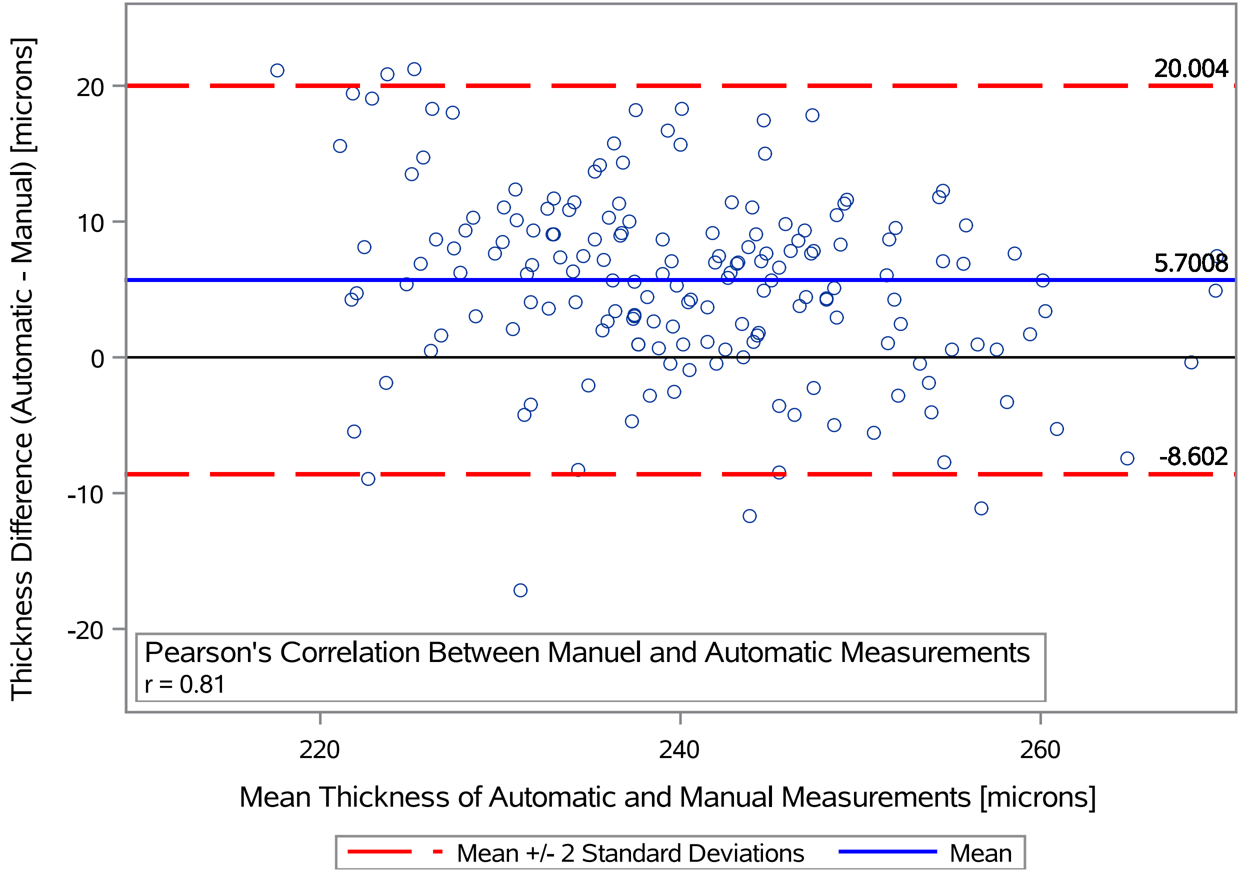

Supplementary Figure S4. Bland-Alman plot showing the differences between retinal thicknesses measured by the automatic and manual techniques. Mean difference (blue line) and two standard deviations (dashed red lines) are plotted with all differences (blue circle). Pearson's correlation statistics revealed a correlation factor of 0.82 between the two techniques with a maximum difference of 20.0 μm (90% two-sided confidence intervals).
