# Supplementary material for: Morphometric and Microstructural Changes During Murine Retinal Development Characterized Using In Vivo Optical Coherence Tomography
Source: Invest Ophthalmol Vis Sci. 2021 Oct 26;62(13):20. doi: 10.1167/iovs.62.13.20 (PMC8556565; doi:10.1167/iovs.62.13.20)
Supplement: Supplement 6 [file iovs-62-13-20_s006.pdf]

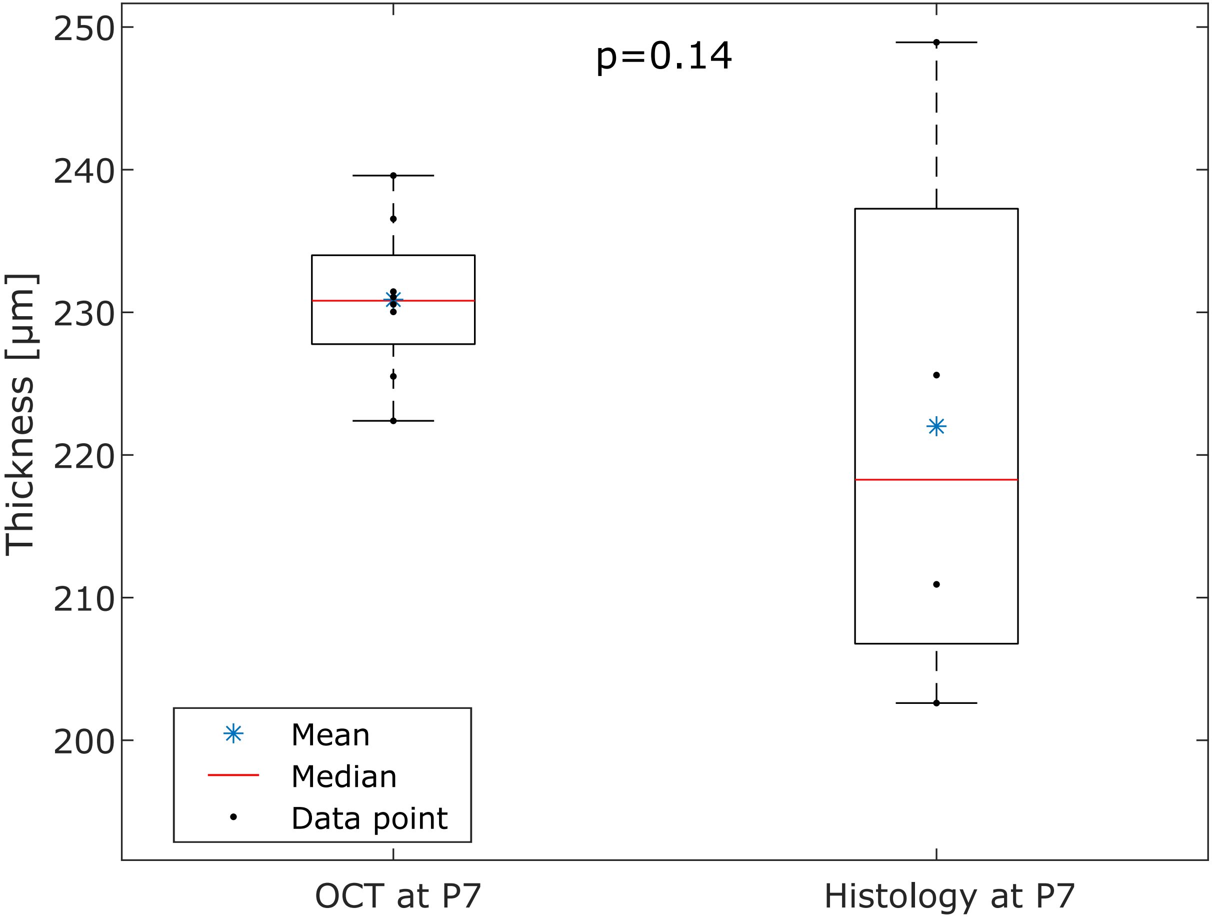

Supplementary Figure S6. Comparison between *in vivo* OCT and histology for retinal thicknesses measured at P7. A linear mixed model with repeated measurements was used to show that OCT and histological measurements were not significantly different at P7 ($p = 0.14$).
