# Supplementary material for: Morphometric and Microstructural Changes During Murine Retinal Development Characterized Using In Vivo Optical Coherence Tomography
Source: Invest Ophthalmol Vis Sci. 2021 Oct 26;62(13):20. doi: 10.1167/iovs.62.13.20 (PMC8556565; doi:10.1167/iovs.62.13.20)
Supplement: Supplement 7 [file iovs-62-13-20_s007.pdf]

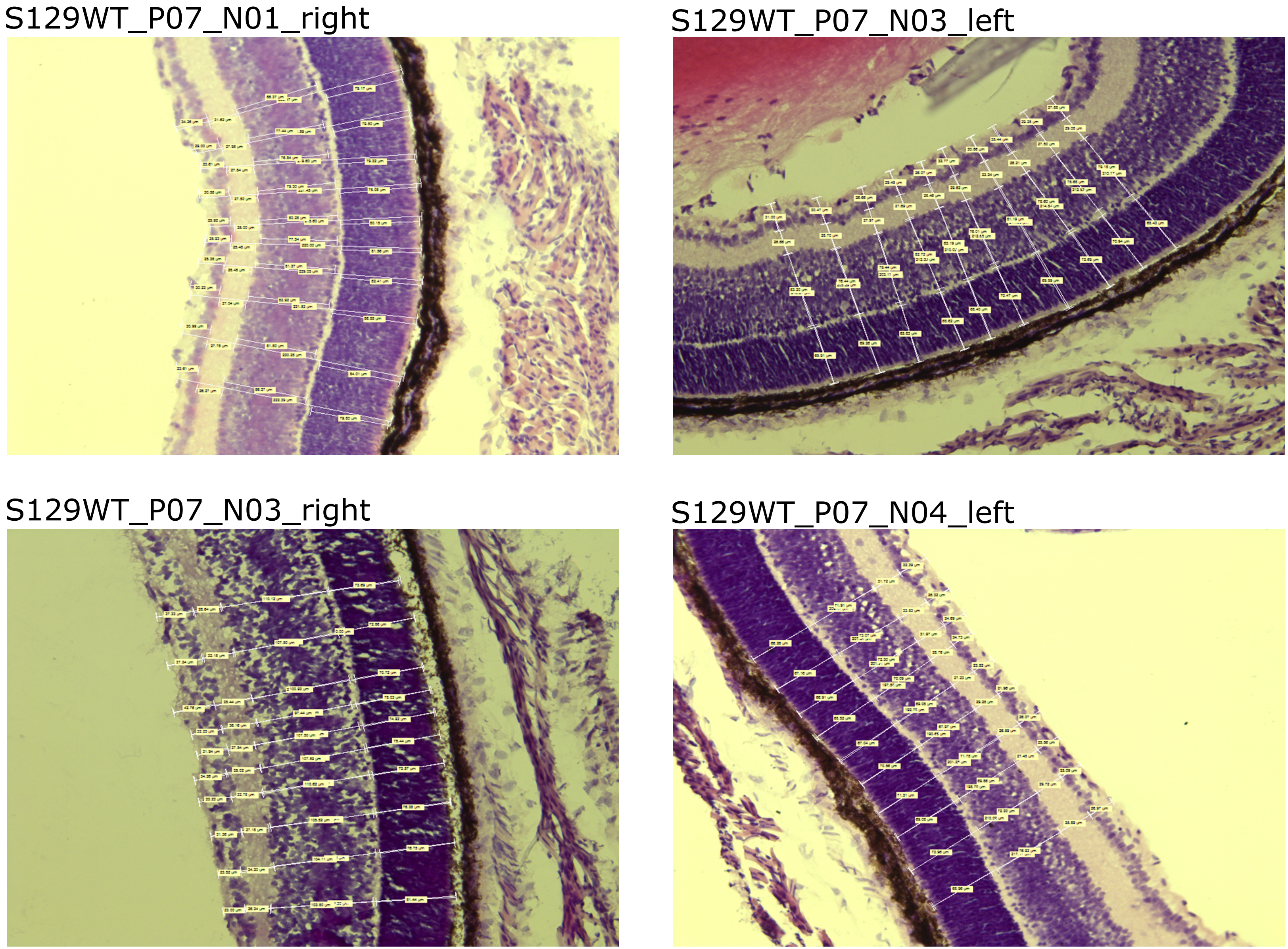

Supplementary Figure S7. Histological images that were used for manual retinal thickness measurements at P7 for validation of the segmentation tool (see values in Supplementary Figure S6).
