# Supplementary material for: Morphometric and Microstructural Changes During Murine Retinal Development Characterized Using In Vivo Optical Coherence Tomography
Source: Invest Ophthalmol Vis Sci. 2021 Oct 26;62(13):20. doi: 10.1167/iovs.62.13.20 (PMC8556565; doi:10.1167/iovs.62.13.20)
Supplement: Supplement 8 [file iovs-62-13-20_s008.pdf]

Supplementary Table S1. The segmentation approach for each retinal layer and their specific identification criteria.

Step	Boundary	Feature	Mask	Criteria
**1**	Retinal pigment epithelium (RPE) estimate	Highest intensity	-	Max(I(z))
**2**	Vitreous to Nerve Fiber Layer (NFL)	High first derivative	Hann filter, 200-250µm above Step 1	Max(dI/dz times Mask)
**3**	Outer Plexiform Layer (OPL)	Higher intensity compared to INL and photoreceptors	Squared Hann filter, between Step 2 and 1	Max(I(z) times Mask)
**4**	Inner Plexiform Layer (IPL) to Inner Nuclear Layer (INL)	Negative first derivative, excluding end of NFL	Linear increase from Step 2 to 3 to exclude end of NFL	Min(dI/dz times Mask)
**5**	NFL to INL	Negative first derivative	Hann filter, between Step 2 and 4	Min(dI/dz times Mask)
**6**	Inner to Outer Segment of the photoreceptor	High first derivative	Hann filter, between Step 3 and 1	Max(I(z) times Mask)

Note: I(z) is the intensity in dB of the image for every point in a A-line. dI/dz is the first derivative along the z direction (depth). The Mask is applied on every A-line independently. The mask in which the boundaries are identified was important as boundaries were used to identify other interfaces by iteration.
