# Supplementary material for: Morphometric and Microstructural Changes During Murine Retinal Development Characterized Using In Vivo Optical Coherence Tomography
Source: Invest Ophthalmol Vis Sci. 2021 Oct 26;62(13):20. doi: 10.1167/iovs.62.13.20 (PMC8556565; doi:10.1167/iovs.62.13.20)
Supplement: Supplement 9 [file iovs-62-13-20_s009.pdf]

Supplementary Table S2. Statistical comparisons of retinal layer thicknesses between all timepoints (from postnatal (P) day 7 to P21) using linear mixed models with repeated measurements. A Tukey-Kramer correction was applied to account for multiple comparisons and a corrected *p*-value < 0.05 was considered significant.

**Nerve Fiber Layer (NFL)**						
**Age [days]**	**P7**	**P10**	**P12**	**P14**	**P17**	**P21**
**P7**	N/A	N/A	N/A	N/A	N/A	N/A
**P10**	n.s.	N/A	N/A	N/A	N/A	N/A
**P12**	n.s.	n.s.	N/A	N/A	N/A	N/A
**P14**	n.s.	n.s.	n.s.	N/A	N/A	N/A
**P17**	n.s.	n.s.	n.s.	n.s.	N/A	N/A
**P21**	n.s.	n.s.	n.s.	n.s.	n.s.	N/A

**Inner Plexiform Layer (IPL)**						
**Age [days]**	**P7**	**P10**	**P12**	**P14**	**P17**	**P21**
**P7**	N/A	N/A	N/A	N/A	N/A	N/A
**P10**	**0.0002**	N/A	N/A	N/A	N/A	N/A
**P12**	**< 0.0001**	**< 0.0001**	N/A	N/A	N/A	N/A
**P14**	**< 0.0001**	**< 0.0001**	n.s.	N/A	N/A	N/A
**P17**	**< 0.0001**	**< 0.0001**	n.s.	n.s.	N/A	N/A
**P21**	**< 0.0001**	**< 0.0001**	n.s.	n.s.	n.s.	N/A

**Inner Nuclear Layer (INL)**						
**Age [days]**	**P7**	**P10**	**P12**	**P14**	**P17**	**P21**
**P7**	N/A	N/A	N/A	N/A	N/A	N/A
**P10**	**0.0033**	N/A	N/A	N/A	N/A	N/A
**P12**	**< 0.0001**	**< 0.0001**	N/A	N/A	N/A	N/A
**P14**	**< 0.0001**	**< 0.0001**	n.s.	N/A	N/A	N/A
**P17**	**< 0.0001**	**< 0.0001**	**0.0017**	n.s.	N/A	N/A
**P21**	**< 0.0001**	**< 0.0001**	**< 0.0001**	**< 0.0001**	**0.0001**	N/A

**Outer Retinal Layer (ORL)**						
**Age [days]**	**P7**	**P10**	**P12**	**P14**	**P17**	**P21**
**P7**	N/A	N/A	N/A	N/A	N/A	N/A
**P10**	n.s.	N/A	N/A	N/A	N/A	N/A
**P12**	**< 0.0001**	**< 0.0001**	N/A	N/A	N/A	N/A
**P14**	**< 0.0001**	**< 0.0001**	n.s.	N/A	N/A	N/A
**P17**	**< 0.0001**	**< 0.0001**	**0.0163**	n.s.	N/A	N/A
**P21**	**< 0.0001**	**< 0.0001**	**0.0002**	**0.0352**	n.s.	N/A

**Total Retina**						
**Age [days]**	**P7**	**P10**	**P12**	**P14**	**P17**	**P21**
**P7**	N/A	N/A	N/A	N/A	N/A	N/A
**P10**	**0.0163**	N/A	N/A	N/A	N/A	N/A
**P12**	**< 0.0001**	**< 0.0001**	N/A	N/A	N/A	N/A

**P14**	**< 0.0001**	**< 0.0001**	n.s.	N/A	N/A	N/A
**P17**	**< 0.0001**	**< 0.0001**	n.s.	n.s.	N/A	N/A
**P21**	**< 0.0001**	**< 0.0001**	n.s.	n.s.	n.s.	N/A

Note: Non-significant (n.s.); Not applicable (N/A).
