# Supplementary material for: Morphometric and Microstructural Changes During Murine Retinal Development Characterized Using In Vivo Optical Coherence Tomography
Source: Invest Ophthalmol Vis Sci. 2021 Oct 26;62(13):20. doi: 10.1167/iovs.62.13.20 (PMC8556565; doi:10.1167/iovs.62.13.20)
Supplement: Supplement 10 [file iovs-62-13-20_s010.pdf]

Supplementary Table S3. Retinal layer thicknesses for each time-point (from postnatal (P) day 7 to P21). Averages and standard deviations are showed for the nerve fiber layer (NFL), inner plexiform layer (IPL), inner nuclear layer (INL), outer retinal layer (ORL), as well as the total retinal thickness (N = number of retinas).

Age [days]	NFL [µm]	IPL [µm]	INL [µm]	ORL [µm]	Total Retina [µm]
**P7 (N = 8)**	16 ± 1	44 ± 2	60 ± 3	111 ± 4	231 ± 5
**P10 (N = 10)**	17 ± 1	49 ± 2	56 ± 1	116 ± 3	239 ± 4
**P12 (N = 7)**	19 ± 2	56 ± 1	50 ± 2	128 ± 3	253 ± 4
**P14 (N = 14)**	17 ± 1	58 ± 2	48 ± 1	133 ± 3	255 ± 5
**P17 (N = 7)**	17 ± 3	59 ± 1	46 ± 1	135 ± 4	257 ± 5
**P21 (N = 8)**	18 ± 3	56 ± 2	41 ± 2	138 ± 5	253 ± 6
